# Outcomes of patients with hematological malignancies complicated with autoimmune diseases after allogeneic hematopoietic stem cell transplantation: A ten-year real-world study

**DOI:** 10.1515/jtim-2026-0031

**Published:** 2026-06-10

**Authors:** Qiusha Huang, Jin Wu, Haixia Fu, Peng Zhao, Zhuoyu An, Yun He, Xiaolu Zhu, Qi Chen, Fengrong Wang, Yuanyuan Zhang, Xiaodong Mo, Wei Han, Chenhua Yan, Jingzhi Wang, Huan Chen, Yuhong Chen, Tingting Han, Meng Lv, Yao Chen, Yu Wang, Lanping Xu, Xiaojun Huang, Xiaohui Zhang

**Affiliations:** Peking University People's Hospital, Peking University Institute of Hematology, Beijing, China; National Clinical Research Center for Hematologic Disease, Beijing, China; Beijing Key Laboratory of Hematopoietic Stem Cell Transplantation, Beijing, China; Collaborative Innovation Center of Hematology, Peking University, Beijing, China

**Keywords:** autoimmune diseases, hematological malignancies, allogeneic hematopoietic stem cell transplantation

## Abstract

**Background and Objectives:**

Patients with autoimmune diseases (ADs) are at an increased risk of developing hematological malignancies (HMs). While allogeneic hematopoietic stem cell transplantation (allo-HSCT) is a curative option for HMs, the outcomes in patients with concomitant ADs and the effects of transplantation on autoimmune conditions remain poorly characterized.

**Methods:**

In this real-world study, we compared transplantation outcomes between 41 patients with HMs and preexisting ADs and 123 matched controls with HMs but without ADs.

**Results:**

A total of 65.8% of the patients with ADs had a disease duration of ≥ 3 years. All patients achieved hematological recovery. The AD group had a significantly higher incidence of chronic graft-versus-host disease (70.7% *vs*. 26.8%, *P* < 0.001). After a median follow-up of 24 (8–75) months, the 3-year overall survival (73.2% [95% CI: 60.2%–89.1%] *vs*. 76.3% [95% CI: 68.9%–84.4%], *P* = 0.76) and disease-free survival rates (68.8% [95% CI: 55.6%–85.3%] *vs*. 74.7% [95% CI: 67.2%–82.9%], *P* = 0.57) were comparable between the AD and control groups. No significant differences were observed in the 3-year cumulative incidence of relapse (7.7% [95% CI: 1.9%–18.8%] *vs*. 9.5% [95% CI: 5.0%–15.7%], *P* = 0.61) or nonrelapse mortality (23.5% [95% CI: 11.5%–38.0%] *vs*. 16.7% [95% CI: 10.6%–23.9%], *P* = 0.40). Multivariable analysis confirmed that preexisting AD was not an independent risk factor for survival, relapse, or nonrelapse mortality.

**Conclusions:**

Our results suggest that allo-HSCT in patients with HMs and ADs is associated with survival outcomes that appear comparable to those in patients without ADs but also induces complete remission of AD in most patients.

## Introduction

Autoimmune diseases (ADs) arise from misdirected immune responses against self-antigens and affect a significant proportion of the global population.^[1]^ Most ADs currently remain incurable, and require lifelong treatment,^[2,3]^ resulting in a consistently growing disease burden.^[2]^ Notably, patients with ADs such as rheumatoid arthritis (RA) and systemic lupus erythematosus (SLE) are well documented to have an increased risk of developing hematological malignancies (HMs), including leukemias and myelodysplastic syndrome (MDS).^[4, 5, 6]^ This association is attributed to chronic inflammation, immune dysregulation, and the effects of prior immunosuppressive therapies.^[6, 7, 8]^

Despite advances in targeted therapy and immunotherapy, allogeneic hematopoietic stem cell transplantation (allo-HSCT) remains the only curative therapy for various hematological malignancies. Recently, HSCT has emerged as a potent treatment option for severe, refractory ADs.^[9, 10, 11]^ While autologous HSCT has been more widely applied in this setting, and substantial evidence from registries such as the European Society for Blood and Marrow Transplantation (EBMT) support its efficacy in diseases such as multiple sclerosis and systemic sclerosis,^[10,12,13]^ its benefits are often limited by a significant risk of disease relapse, likely due to the persistence of autoreactive lymphocytes.^[14]^ Allo-HSCT offers the potential for cure but has inherent risks of graft-versus-host disease (GVHD) and transplant-related mortality (TRM).

A critical, yet understudied, clinical scenario arises in patients with concurrent HMs and ADs. In these patients, the primary indication for allo-HSCT is hematological malignancy. However, the impacts of preexisting ADs on transplant outcomes—including engraftment, GVHD, relapse, and nonrelapse mortality—remain poorly defined. Furthermore, whether allo-HSCT can concurrently induce remission of concomitant AD is poorly understood. Data specifically addressing this comorbid population are limited.

To address this issue, we conducted a retrospective study to investigate post-allo-HSCT outcomes in patients with HMs and preexisting ADs. We aimed to determine (1) the prognostic impacts of preexisting ADs on posttransplant survival and relapse and (2) the efficacy of allo-HSCT in inducing sustained remission of the concomitant autoimmune condition.

## Materials and methods

### Patients and study design

This retrospective study reviewed data from 8596 patients who underwent allo-HSCT from May 2012 to April 2022 at the Peking University Institute of Hematology. We used the International Classification of Diseases (ICD)-10-Clinical Modification (CM) and ICD-11-CM codes to retrieve data of allo-HSCT patients who were diagnosed with Behçet's disease (BD), SLE, RA and other ADs. Patients with more than one documented AD were described collectively in baseline tables. To ensure that each patient fulfilled the diagnostic criteria for each AD, a hematologist and a rheumatologist jointly reviewed the medical records of every patient. Finally, 41 hematological malignancy patients with concomitant ADs were identified. In this real-world study, we compared transplantation outcomes after allo-HSCT between 41 patients with HMs and preexisting ADs and 123 matched controls with HMs but without ADs.

**Figure 1:**
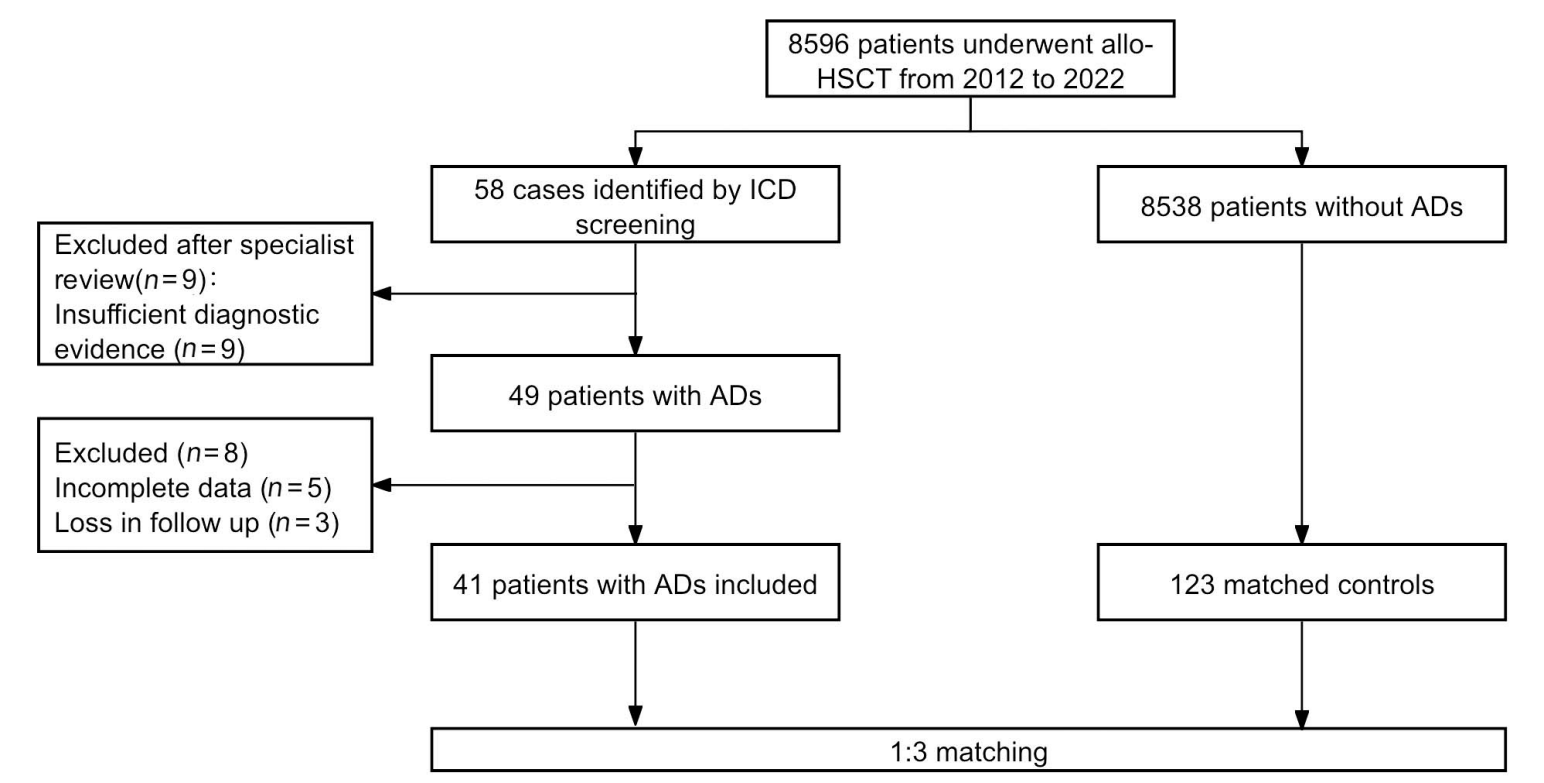
Algorithm of study design and patient selection. AD: autoimmune disease; allo-HSCT: allogeneic hematopoietic stem cell transplantation; ICD: International Classification of Diseases.

We conducted a real-world study to compare the outcomes among patients with and without ADs. For each patient with AD, 3 controls were randomly matched without replacement for the primary disease, transplantation type, conditioning regimen, sex, and age (±3 years). These variables were prioritized for matching as they represent structural factors that define the transplant framework. This matching strategy ensured comparability in key prognostic baseline characteristics between the AD and non-AD groups. This matching strategy ensured comparability in key prognostic baseline characteristics between the AD and non-AD groups. Finally, 41 patients with ADs and 123 patients without ADs were included in our study (Figure 1).

The study was approved by the ethics committee of Peking University People’s Hospital (2025PHB073). The study conforms to the principles of the Declaration of Helsinki. Informed consent was obtained from all the patients.

### Definitions

Hematological malignancies are a group of blood diseases that originate from the clonal expansion of hematopoietic cells. The main types are leukemia, MDS, myeloproliferative neoplasms (MPNs), multiple myeloma and lymphoma.^[15]^ We investigated 17 of the most common ADs that are treated with HSCT from the EBMT database, namely, inflammatory bowel disease (Crohn's disease or ulcerative colitis), type 1 diabetes, MS, myasthenia gravis, polymyalgia rheumatica (PMR), RA, juvenile idiopathic arthritis (JIA), ankylosing spondylitis (AS), psoriatic arthritis, Sjögren's syndrome (SS), SLE, antiphospholipid syndrome (APS), systemic sclerosis, polymyositis (PM), dermatomyositis (DM), and vasculitis.^[11]^ The detailed ICD-10 and ICD-11 codes are listed in Supplementary Table S1. The detailed classification criteria are provided in Supplementary Table S2.

AD responses were assessed on the basis of the following criteria. Complete remission (CR) post-HSCT was defined as the absence of clinical or laboratory evidence of AD activity.^[14,16]^ Partial remission (PR) was defined as (1) a significant improvement in clinical symptoms and the cessation of immunosuppression or (2) complete resolution of inflammatory symptoms with ongoing immunosuppression. No remission (NR) was defined as a failure to achieve a PR or CR at any time point.^[14,16]^ The determination of AD remission status for this study was made through a retrospective review conducted in collaboration with a consulting rheumatologist. The rheumatologist reviewed all available post-transplant clinical documentation.

To incorporate an objective laboratory measure, the trend in the erythrocyte sedimentation rate (ESR) was evaluated. White blood cell engraftment was defined as an absolute neutrophil count ≥ 0.5 × 10^9^/L for 3 consecutive days after transplantation. Platelet engraftment was defined as a platelet count ≥ 20 × 10^9^/L without platelet transfusion for 7 consecutive days. Acute GVHD (aGVHD) and chronic GVHD (cGVHD) were assessed according to previously described clinical criteria.^[17,18]^

### Conditioning regimens and GVHD prophylaxis

All patients in this cohort underwent allo-HSCT exclusively on the basis of standard indications for their hematological malignancies and not for the treatment of their ADs. Most patients received Bu/Cy/anti-human thymocyte immunoglobulin (ATG) or Bu/ Cy myeloablative conditioning regimens.^[19, 20, 21, 22, 23]^ Most patients received allografts from donors with partial HLA matching. These patients received ATG-based haploidentical HSCT following a previously established protocol.^[19,20,24]^ All of the transplant recipients received mycophenolate mofetil (MMF), cyclosporine A (CsA), and short-term methotrexate (MTX) for GVHD prophylaxis.^[25,26]^

The protocol for routine post-transplant clinical follow-up at our center was structured as follows: patients were assessed weekly for the first 3 months, biweekly from months 4 to 6, and at least monthly thereafter, or more frequently if clinically indicated. This schedule ensured regular monitoring of both transplant and AD-related symptoms.

### Statistical analysis

Survival curves were generated *via* the Kaplan-Meier method and compared *via* the log-rank test. Nonrelapse mortality (NRM) was defined as death from any cause in the first 28 days or death without evidence of disease relapse beyond day 28. The cumulative incidence of relapse (CIR) and NRM were estimated using competing risks analysis, with death without relapse and relapse treated as mutually exclusive competing events, respectively. Analyses were performed using the "cmprsk" package in R. Endpoint events for disease-free survival (DFS) included morphological relapse and NRM. We did a complete case analysis with no imputation for missing data.

All the statistical analyses were run with R version 4.3.1 and SPSS version 25. Continuous variables are presented as medians with interquartile ranges (IQRs). Categorical variables are presented as counts (%). We compared patient characteristics between cases and controls by using the chi-square (*χ*^2^) test or Fisher's exact test for categorical variables and the Mann-Whitney *U* test for continuous variables. A paired *t* test was used to compare the ESR before and after transplantation. The proportional hazards assumption for Cox models was determined by Kaplan-Meier analysis and was found to be met for all covariates included in the final models. For multivariable Cox proportional hazards models, all variables with a *P* value < 0.1 in univariable analysis were considered for inclusion. A backward stepwise selection procedure with a threshold of *P* < 0.05 was used for the final model. All hazard ratios (HRs) are reported alongside their 95% confidence intervals (CIs) and *P* values to allow for assessment of both clinical impact and statistical precision. To address potential era effects, a sensitivity analysis was performed by categorizing the transplant year into two periods (2012–2016 and 2017–2022) based on case distribution. Survival outcomes were compared between these periods within both the AD and control cohorts (Supplementary Figure S1). Variables that were used for matching (*e.g*., primary disease and sex) are presented descriptively, without formal hypothesis testing, as they were by design balanced between the groups. Statistical significance was defined as a two-sided *P* value < 0.05.

## Results

### Patient characteristics

The baseline characteristics of the patients are listed in Table 1. There were more females (58.5%) than males (41.5%). Most patients with AD (65.9%) had a disease duration of greater than 3 years. The median time from the onset of an AD to the diagnosis of a hematological malignancy was 5 (1–9) years. The proportion of patients with acute leukemia and MDS/MPN was 95.1%, accounting for the majority of the patients included.

**Table 1:** Baseline characteristics of the patients with hematological malignancies with pre-existing AD

Characteristics	Patients with ADs (*n* = 41)
Age at AD diagnosis (years)	33 (21.5-45.5)
Gender	
Male	17 (41.5)
Female	24 (58.5)
AD type	
BD	8 (19.5)
Takayasu’s arteritis	1 (2.4)
PMR	2 (4.9)
SS	2 (4.9)
APS	1 (2.4)
RA	6 (14.6)
AS	14 (34.1)
SLE	4 (9.8)
JIA	1 (2.4)
SLE and APS	1 (2.4)
SS and RA	1 (2.4)
Hematological malignancies	
ALL	12 (29.3)
AML	15 (36.6)
MDS	10 (24.4)
CMML	2 (4.9)
Lymphoma	2 (4.9)
Treatment for AD	
No treatment	5 (12.2)
Glucocorticoids only	4 (9.8)
Immunosuppressants/immuno-modulators only	6 (14.6)
NSAIDs only	9 (22)
Combination therapy without biologics	14 (34.1)
Therapy including biologics	3 (7.3)
AD duration (years)	
< 3	14 (34.1)
3-10	21 (51.2)
> 10	6 (14.6)

Data are presented as *n* (%) or median (IQR). allo-HSCT: allogeneic hematopoietic stem cell transplantation; AD: autoimmune disease; AML: acute myeloid leukemia; ALL: acute lymphoblastic leukemia; MDS: myelodysplastic syndrome; BD: Behcet’s diseases; PMR: polymyalgia rheumatica; SS: Sjogren’s syndrome; APS: antiphospholipid syndrome; RA: rheumatoid arthritis; AS: ankylosing spondylitis; SLE: systemic lupus erythematosus; JIA: juvenile idiopathic arthritis; NSAIDs: non-steroidal antiinflammatory drugs; IQR: interquartile range.

### Transplant outcomes

The patient characteristics are summarized in Table 2. For pre-transplant AD medication, glucocorticoids were tapered and, when possible, discontinued prior to the initiation of conditioning therapy. While immunosuppressants/ immunomodulators or biologics were discontinued according to their pharmacokinetic profile and transplant regimen requirements. The median mononuclear cells (MNC) dose was marginally higher in the AD group (8.64 × 10^8^/kg *vs*. 8.21 × 10^8^/kg, *P* = 0.050); however, the CD34 cell dose, a more direct and critical determinant of hematopoietic engraftment, showed no difference between the groups. All patients achieved hematological recovery (Table 3). The median time to white blood cell engraftment was 14 (12–15) days, whereas the median time to platelet engraftment was 17.0 (13.0–19.5) days. The patients with ADs required a longer time to Platelet (PLT) engraftment than the controls did (*P* = 0.020). Twenty-four patients (58.5%) experienced grade I–IV acute GVHD, and 29 patients (70.7%) experienced chronic GVHD. The patients with ADs experienced more cGVHD than the controls did (*P* < 0.001). The detailed characteristics of cGVHD are presented in Supplementary Table S3. As shown in Supplementary Figure S2, the occurrence of cGVHD was not associated with a statistically significant increase in NRM, CIR or worse survival. Twenty-six (63.4%) patients experienced Cytomegalovirus (CMV) infection, and 6 (14.6%) patients experienced Epstein-Barr virus (EBV) infection. No statistical differences were observed in CMV and EBV infection between the two groups.

**Table 2:** Baseline characteristics of the patients with pre-existing AD and matched controls after allo-HSCT

Characteristics	Patients with ADs (*n* = 41)	Controls (*n* = 123)	*P*
Age at HSCT (years)	42.0 (26.5-52.5)	42.0 (28.0-49.0)	0.805
Gender			-
Male	17 (41.5)	51 (41.5)	
Female	24 (58.5)	72 (58.5)	
Primary disease			-
ALL	12 (29.3)	36 (29.3)	
AML	15 (36.6)	45 (36.6)	
MDS	10 (24.4)	30 (24.4)	
Others	4 (9.8)	12 (9.8)	
ABO matched			0.367
Match	24 (58.5)	62 (50.4)	
Mismatch	17 (41.5)	61 (49.6)	
Stem cell source			0.381
BM + PB	26 (63.4)	87 (70.7)	
PB	15 (36.6)	36 (29.3)	
Type of transplantation			-
Haplo-identical related	30 (73.2)	90 (73.2)	
HLA match related	8 (19.5)	24 (19.5)	
Unrelated match	3 (7.3)	9 (7.3)	
Conditioning regimen			-
Bu/Cy	6 (14.6)	18 (14.6)	
Bu/Cy+ATG	22 (53.7)	66 (53.7)	
Others	13 (31.7)	39 (31.7)	
MNC (× 10^8^/kg)	8.64 (7.74-10.38)	8.21 (7.29-9.25)	0.050
CD34^+^ (× 10^6^/kg)	3.24 (1.82-4.27)	2.92 (1.97-4.31)	0.717

Data are presented *n* (%) or median (IQR). The matching variables (sex, primary disease, type of transplantation, and conditioning regimen) are indicated by a dash (-) for the *P* value. allo-HSCT: allogeneic hematopoietic stem cell transplantation; AD: autoimmune disease; AML: acute myeloid leukemia; ALL: acute lymphoblastic leukemia; MDS: myelodysplastic syndrome; HSCT: hematopoietic stem cell transplantation; BM: bone marrow; PB: peripheral blood; Bu: busulfan; Cy: cytarabine; ATG: antithymocyte globulin; MNC: mononuclear cell; IQR: interquartile range.

**Table 3:** Transplant outcome of the patients with pre-existing AD and matched controls after allo-HSCT

Characteristics	Patients with ADs (*n* = 41)	Controls (*n* = 123)	*P*
WBC engraftment (days)	14 (12-15)	13 (12-15)	0.265
PLT engraftment (days)	17.0 (13.0-19.5)	13.0 (11.0-18.0)	0.020
Acute GVHD			0.071
None	17 (41.5)	75 (61.0)	
I-II	20 (48.8)	37 (30.1)	
III-IV	4 (9.8)	11 (8.9)	
Chronic GVHD			< 0.001
None	12 (29.3)	90 (73.2)	
Limited	23 (56.1)	19 (15.4)	
Extensive	6 (14.6)	14 (11.4)	
CMV viremia	26 (63.4)	70 (56.9)	0.464
EBV viremia	6 (14.6)	10 (8.1)	0.233

Data are presented as median (IQR) or *n* (%). allo-HSCT: allogeneic hematopoietic stem cell transplantation; AD: autoimmune disease; WBC: white blood cell; PLT: platelet; GVHD: graft versus host disease; CMV: cytomegalovirus; EBV: Epstein-Barr virus; IQR: interquartile range.

**Figure 2:**
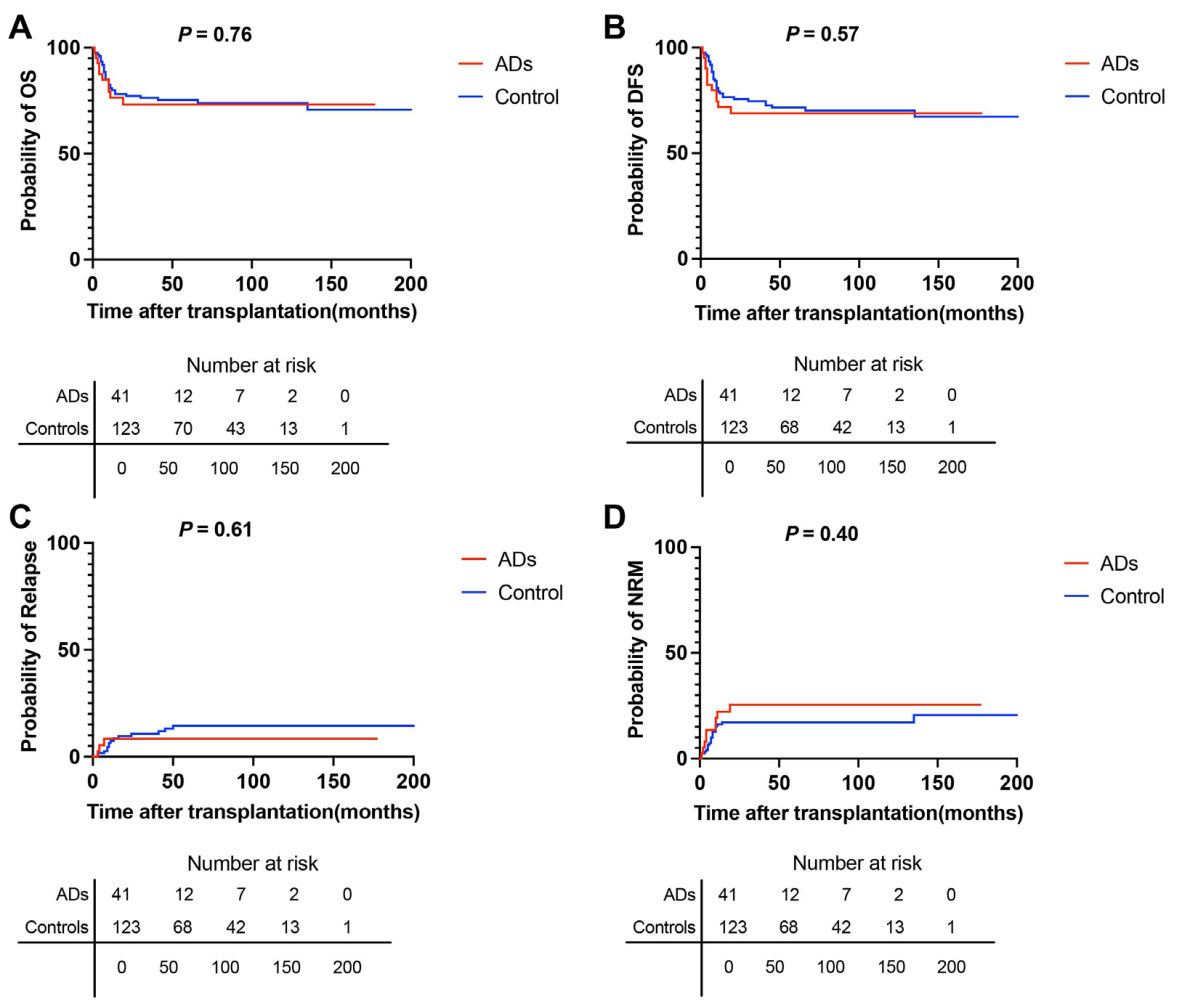
Outcomes after transplantation among patients with hematological malignancies with pre-existing AD and those without AD. The rate of (A) OS and (B) DFS. (C) The incidence of relapse. (D) The incidence of NRM. AD: autoimmune disease; OS: overall survival; DFS: disease free survival; NRM: nonrelapse mortality.

We compared the outcomes among patients with and without ADs (Figure 2). The median follow-up duration was 24 (8–75) months after transplantation. The 3-year DFS rates of patients with and without preexisting ADs were 68.8% (95% CI: 55.6%–85.3%) and 74.7% (95% CI: 67.2%–82.9%) (*P* = 0.57), respectively; the 3-year overall survival (OS) rates were 73.2% (95% CI: 60.2%–89.1%) and 76.3% (95% CI: 68.9%–84.4%) (*P* = 0.76), respectively, and the difference was not significant.

The 3-year CIRs were 7.7% (95% CI: 1.9%–18.8%) and 9.5% (95% CI: 5.0%–15.7%) (*P* = 0.61) among the patients with and without ADs, respectively. Ten patients in the AD group and 31 patients in the control group died. Among the 10 patients with ADs, 1 died of hemoptysis, 4 died from severe pneumonia, 2 died from thrombotic microangiopathy, 1 died from gastrointestinal hemorrhage, 1 died from central nervous system infection, and 1 died from sinusoidal obstruction syndrome. The 3-year cumulative incidences of NRM were 23.5% (95% CI: 11.5%–38.0%) and 16.7% (95% CI: 10.6%–23.9%) (*P* = 0.40) among the patients with and without ADs, respectively.

We identified a subset of 113 patients (including 23 AD patients and 90 control patients) who either experienced an event (death or relapse) within 5 years or had a follow-up duration of ≥ 5 years. In this cohort, the 5-year OS was 54.2% (95% CI: 36.7%–80.1%) for the AD group versus 67.6% (95% CI: 58.6%–78.0%) for controls (*P* = 0.23), and 5-year DFS was 47.8% (95% CI: 31.2%–73.3%) *vs*. 63.3% (95% CI: 54.1%–74.1%) (*P* = 0.12). The 5-year CIR was 13.0% (95% CI: 3.1%–30.2%) *vs*. 15.7% (95% CI: 9.1%–24.1%) (*P* = 0.82), and 5-year NRM was 39.1% (95% CI: 19.4%–58.5%) *vs*. 22.2% (95% CI: 14.3%–31.3%) (*P* = 0.12). These results demonstrate that the comparable survival outcomes between groups observed at 2 years are maintained at the 5-year landmark.

We compared transplant outcomes by pre-transplant AD activity (active *vs*. stable) and AD duration (< 3 years *vs*. ≥ 3 years). The analysis did not reveal statistically significant differences in key outcomes between these subgroups within the AD cohort (Supplementary Figure S3 and S4).

Univariable and multivariable analyses of prognostic factors were performed (Supplementary Table S4–7). Grade 3–4 aGVHD and CMV infection were associated with higher NRM and lower OS and DFS. Multivariable analysis also revealed no associations between ADs and OS, DFS, the CIR, or NRM.

### Post-transplant AD remission outcomes

**Figure 3:**
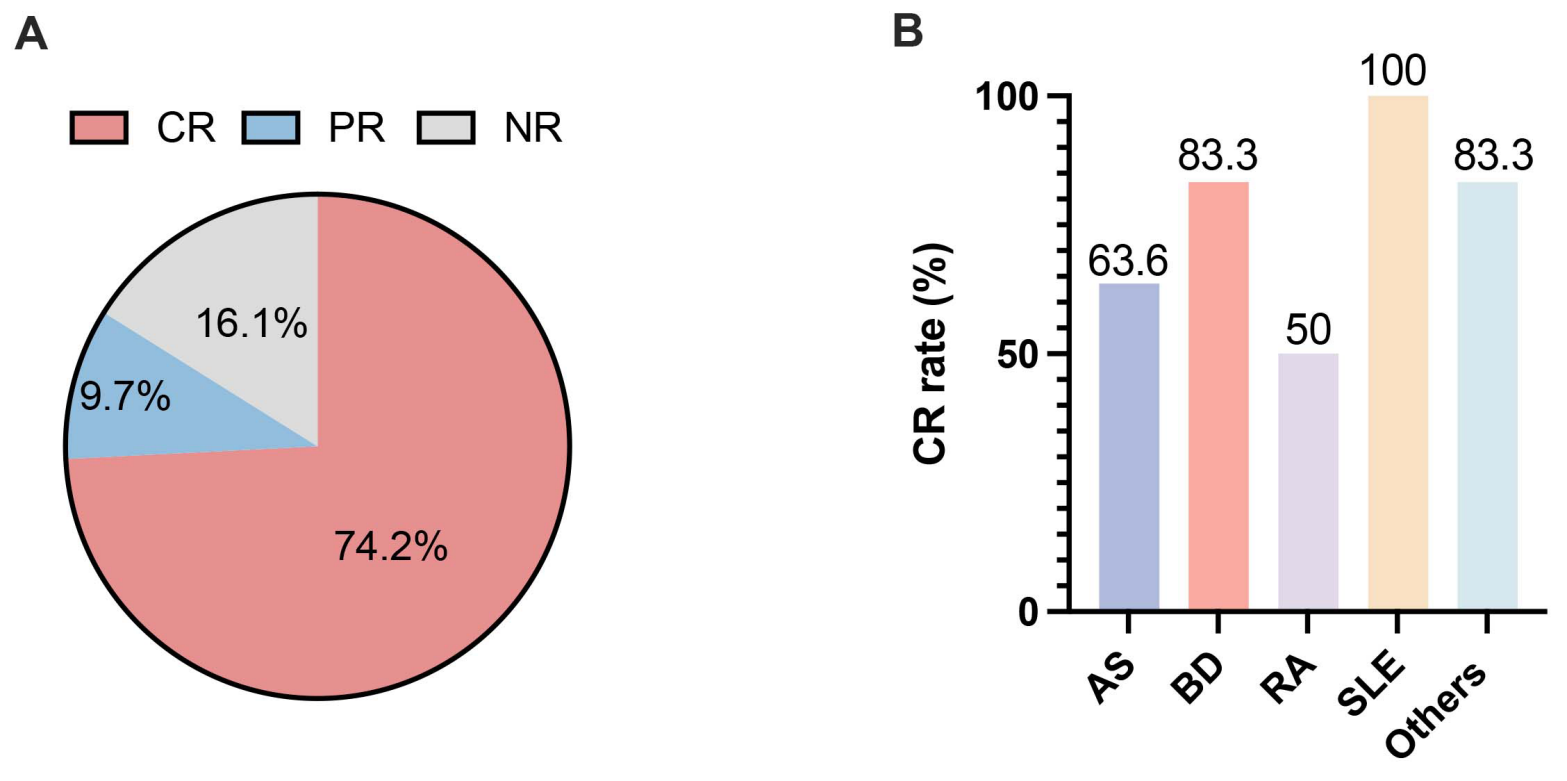
Remission outcomes of pre-existing ADs after allo-HSCT stratified by major subtypes. (A) Pie chart showing the remission status of ADs at last follow-up in surviving patients (*n* = 31). (B) Bar graph depicting complete remission rate stratified by major AD subtypes. AD: autoimmune disease; CR: complete remission; PR: partial remission; NR: no remission; BD: Behcet's diseases; RA: rheumatoid arthritis; AS: ankylosing spondylitis; SLE: systemic lupus erythematosus; ESR: erythrocyte sedimentation rate; HSCT: hematopoietic stem cell transplantation.

At the last follow-up, among the 31 surviving patients evaluable for AD remission status, 23 (74.2%) were in CR, 3 (9.7%) were in PR, and 5 (16.1%) had NR. Among the major AD subtypes, CR rates were 63.6% (AS), 83.3% (BD), 50% (RA), 100% (SLE), and 83.3% (others), respectively (Figure 3). Individual patient characteristics and outcome were shown in Supplementary Table S8. Among the 23 patients who achieved CR, the median time from transplant to CR was 6 (6–8) months. The median time to CR across AD types were listed in Supplementary Table S9. The median duration of CR was 24 (5–59) months. Three patients (1 RA, 1 BD, 1 AS) achieved PR and experienced significant improvements in clinical symptoms at the final follow-up, but they were still dependent on immunosuppression. One patient with BD experienced a relapse of BD 2 years post-HSCT. This patient was managed with prednisone (20 mg daily) combined with azathioprine, and the symptoms were subsequently controlled. Two patients experienced hematologic relapse; their ADs achieved a CR posttransplantation, and no AD relapse was observed at the last follow-up.

**Figure 4:**
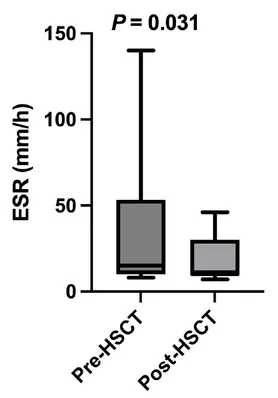
ESR pre- and post-HSCT. Data were available for 29 patients at both pre- and post-HSCT timepoints. ESR: erythrocyte sedimentation rate; HSCT: hematopoietic stem cell transplantation; CR: complete remission; PR: partial remission; NR: no remission; AS: ankylosing spondylitis; BD: Behcet's diseases; RA: rheumatoid arthritis; SLE: systemic lupus erythematosus.

The ESR reflects the disease activity of AD. We evaluated the change in ESR before and after allo-HSCT (Figure 4). Data were available for 29 patients at both pre- and post-HSCT timepoints. Although not all patients were evaluated for the ESR following HSCT, the median ESR showed a statistically significant decrease after transplantation (*P* = 0.031).

Next, we examined the association between the development of cGVHD and the achievement of CR of the pre-existing AD. As shown in Supplementary Table S10, among the 31 surviving patients with evaluable AD status, the incidence of cGVHD was not significantly different between those who achieved CR of their AD (60.9%, 14/23) and those who did not (75.0%, 6/8) (*P* = 0.676).

Given the heterogeneity of ADs in our cohort, we performed a descriptive analysis of key transplant outcomes for the major AD subtypes with sufficient patient numbers (AS, *n* = 14; BD, *n* = 8; RA, *n* = 6; Other ADs [including SLE, SS, APS, *etc*.], *n* = 13). The results are summarized in Supplementary Table S11. Overall, transplant-related outcomes such as engraftment times, incidences of severe (grade III-IV) acute GVHD, and viral reactivation rates showed variability across subgroups. Critically, the key longterm efficacy outcomes of 3-year OS (range: 60.0%–77.9%) and DFS (range: 60.0%–72.9%) were broadly comparable across AD types. Other measures, such as the incidence of chronic GVHD, showed greater variation.

## Discussion

In this retrospective real-world study, our data indicate that patients with hematologic malignancies and concomitant ADs who underwent allo-HSCT had outcomes that were not statistically different from those of patients without ADs. Notably, allo-HSCT concurrently induced sustained remission of preexisting AD in the majority (74.2%) of surviving patients.

In ADs, autoantigen recognition leads to cell-mediated and humoral immune responses and triggers a proinflammatory cascade leading to end-organ damage.^[27]^ Hematopoietic stem cell transplantation eradicates an abnormal autoreactive response of the immune system with chemotherapy, and autologous/allo-HSCT is an attractive therapeutic strategy for treating AD because it can rebuild the immune system and return it to normal function.^[28]^ Many clinical studies have shown that the efficacy of allo-HSCT is superior to that of auto-HSCT and that graft-versus-autoimmune effects can rebuild the lymphatic system and thus promote AD remission.^[29,30]^ Notably, the use of HSCT for the treatment of ADs has greatly improved in terms of patient selection, pretreatment protocol selection, and care support.

The observed incidence of cGVHD in our matched control group (26.8%) is at the lower end of the range reported in contemporary literature (which often spans 30%–70%).^[31,32]^ This aligns with recent reports of a decline in cGVHD, exemplified by a 2024 study documenting a 19% risk reduction per 5-year period.^[33]^ Several factors specific to our study may contribute to this rate: (1) our matching strategy defined a cohort with a particular risk profile; (2) the cGVHD incidence, based on a 24-month median follow-up, may not capture very late-onset cGVHD; and (3) it reflects contemporary improvements in supportive care and prophylaxis.

A primary concern in using transplantation in this population is whether the underlying immune dysregulation increases the risk of transplant-related complications in patients with ADs. While our study revealed no significant difference in the cumulative incidence of relapse or NRM, we observed a higher incidence of chronic GVHD in the AD group. The increased chronic GVHD in AD patients may reflect a shared basis of immune dysregulation. Key overlapping mechanisms likely include: (1) Dysregulated T-cell homeostasis, where pre-existing imbalances between effector and regulatory T-cells could lower the threshold for post-transplant alloreactivity;^[34,35]^ (2) Pathogenic B-cell and antibody responses, where autoantibodies and activated B-cells may synergize with donor immune cells to drive fibrotic cGVHD;^[36,37]^ and (3) a pro-inflammatory tissue microenvironment primed by chronic AD-related cytokines, which can amplify tissue damage and donor cell activation after transplant.^[38]^ However, the similar NRM rates are reassuring and suggest that transplant toxicity is not substantially amplified by the presence of an AD. The comparable survival outcomes held true even after adjusting for known prognostic factors in multivariable analysis, strengthening the conclusion that the AD status itself is not an independent determinant of post-HSCT survival.

The patients with ADs required a longer time to PLT engraftment than the controls did. The observed delay in platelet recovery in AD patients, despite comparable rates of complications like CMV reactivation, likely points to AD-specific factors: (1) Immune-mediated consumption. A key consideration is that patients with ADs may have a baseline state of immune dysregulation. Pre-existing or post-transplant immune complexes, autoantibodies with potential cross-reactivity, or a generalized proinflammatory state could lead to increased peripheral platelet consumption or destruction, thereby delaying the recognition of stable engraftment; (2) Impact of prior therapies. Many patients with ADs receive long-term immunomodulatory medications (*e.g*., corticosteroids, methotrexate) prior to HSCT. Residual effects of these agents on the bone marrow niche or on megakaryocyte progenitor cells may potentially impair platelet production and recovery post-transplant.

Large registry studies, such as the EBMT analysis on allo-HSCT for ADs, primarily include patients who underwent transplantation for the AD itself, often excluding those with concurrent malignancies.^[16]^ Our study directly focused on this underreported "double-hit" population facing the dual challenge of a hematological malignancy and a pre-existing AD. The feasibility and efficacy of allo-HSCT that we observed are consistent with those of smaller case reports and case series,^[39,40]^ but our study provides a more robust, controlled comparison. The similar outcomes to those of patients without ADs reinforce the conclusion that transplant decisions should be driven by the hematologic mandate, with the potential for AD remission as a significant secondary benefit.

The clinical remission of pre-existing AD observed in 74.2% patients is likely attributable to a dual mechanism. First, the allogeneic transplant induces a fundamental immune reconstitution through donor-derived reconstitution. Second, the ongoing immunosuppressive therapy required for GVHD prophylaxis or treatment (*e.g*., calcineurin inhibitors, corticosteroids) concurrently suppresses the pathogenic activity of the AD. Thus, clinical remission likely results from the combined effect of transplant-mediated immune reset and post-transplant pharmacologic suppression.

We acknowledge several important limitations in our study. First, the sample size, while substantial for this rare patient population, remains modest. This limits the statistical power to detect small to moderate differences in outcomes and prevents meaningful subgroup analyses across the spectrum of ADs. We are adequately powered to detect only large effect sizes in major outcomes such as OS. Therefore, the interpretation of negative findings, particularly for secondary endpoints such as NRM or relapse, should be cautious, as the study may be underpowered to identify more modest but clinically relevant differences. Consequently, our study may be underpowered to identify a true association if one exists (Type II error). Therefore, the finding of no significant difference in survival should be interpreted as the absence of a detected effect within the constraints of our study's power, rather than as definitive evidence of equivalence. Second, as a single-center retrospective study conducted over a 10-year period, our findings may be influenced by center-specific protocols and temporal shifts in clinical practice, supportive care, and diagnostic criteria. Consequently, the generalizability of our results to other institutions or more recent treatment eras should be considered with caution. Our study cohort encompasses a spectrum of ADs (*e.g*., AS, BD, RA, SLE) with distinct immunopathologies and clinical courses. This inherent heterogeneity is a fundamental limitation. The sample size within each AD category was too small for statistical subgroup analysis, as presented in our descriptive overview. While this reflects a real-world population, it limits our ability to draw conclusions about specific diseases. The median follow-up of 24 months is a key limitation, as it may be insufficient to capture late-occurring events such as relapse or non-relapse mortality beyond 2 years, the full long-term morbidity of chronic GVHD, and the delayed reactivation of AD. Consequently, our conclusions regarding long-term survival and sustained disease control are primarily applicable to this intermediate-term period, highlighting the need for studies with extended observation.

The assessment of AD activity post-HSCT has limitations. The definition of remission relied on clinical evaluation and immunosuppression use, as standardized disease-specific indices (*e.g*., SLEDAI, DAS28) were not routinely available in this retrospective cohort. While we incorporated ESR analysis as an objective inflammatory marker, which showed a significant post-transplant decrease, these data were not available for all patients, and ESR represented a rather crude overall measure. This study only includes patients who were deemed eligible for allo-HSCT. This introduces a significant selection bias, as patients with highly active, refractory, or severe organ-damaging ADs that contraindicated transplantation are not represented. Consequently, our findings may overestimate the general success rate and underestimate the risks of allo-HSCT for the broader, unselected population of patients with hematological malignancies and comorbid AD.

Due to the nature of the comorbidity under investigation, patients in the AD group had a documented history of immune dysregulation for a median of 5 years prior to their hematologic malignancy diagnosis and transplant. The matched control group had no such history. This introduces an immortal time bias. Although our primary analysis uses transplant date as the common time-zero, the potential influence of this prolonged, pre-existing immunologic background on post-transplant outcomes—compared to patients without it—must be considered when interpreting the results.

Certain established prognostic factors for transplant outcomes, such as the disease risk index and hematopoietic cell transplantation-specific comorbidity index, were not included as matching variables due to our priority to balance the foundational treatment framework within a limited sample size. Their absence from the matching algorithm may result in residual confounding. This is an acknowledged limitation of our cohort matching strategy.

## Conclusion

This study provides evidence that allo-HSCT is an effective strategy for patients with hematologic malignancies and concomitant ADs. It was associated with long-term control of the malignancy and remission of AD in most patients, without detected significant detriment to survival outcomes compared to transplant recipients without AD in our study. Future prospective studies are warranted to validate these findings.

## Supplementary Material

Supplementary Material Details
