# Supplementary material for: Outcomes of patients with hematological malignancies complicated with autoimmune diseases after allogeneic hematopoietic stem cell transplantation: A ten-year real-world study
Source: J Transl Int Med. 2026 Jun 10;14(4):579–88. doi: 10.1515/jtim-2026-0031 (PMC13509153; doi:10.1515/jtim-2026-0031)
Supplement: Supplementary file 1 [file jtim-2026-0031_sm.pdf]

# Supplementary materials

## Outcomes of patients with hematological malignancies complicated with autoimmune diseases after allogeneic hematopoietic stem cell transplantation: A ten-year real-world study

### Table of contents


| | |
|---|---|
| Supplementary Table S1. The ICD codes of included ADs in our study | **2** |
| Supplementary Table S2. Classification criteria of autoimmune diseases | **3** |
| Supplementary Table S3. Characteristics of cGVHD in patients with pre-existing AD | **9** |
| Supplementary Table S4. Univariable and multivariable analysis of prognostic factors for OS | **9** |
| Supplementary Table S5. Univariable and multivariable analysis of prognostic factors for DFS | **10** |
| Supplementary Table S6. Univariable and multivariable analysis of risk factors for NRM | **10** |
| Supplementary Table S7. Univariable and multivariable analysis of risk factors for CIR | **10** |
| Supplementary Table S8. Individual patient characteristics and outcome | **12** |
| Supplementary Table S9. Time to complete remission across different AD types | **16** |
| Supplementary Table S10. Association between the development of cGVHD and the achievement of complete remission of the pre-existing AD | **16** |
| Supplementary Table S11. Transplant outcomes across major autoimmune disease subtypes | **16** |
| Supplementary Figure S1. Comparison of survival probabilities stratified by transplant year period (2012-2016 *vs.* 2017-2022) | **17** |
| Supplementary Figure S2. Association between chronic GVHD and key transplant outcomes in patients with hematological malignancies with pre-existing AD | **17** |
| Supplementary Figure S3. Association between ADs severity and key transplant outcomes in patients with hematological malignancies with pre-existing AD (active *vs.* stable) | **18** |
| Supplementary Figure S4. Association between ADs duration and key transplant outcomes in patients with hematological malignancies with pre-existing AD (disease duration < 3 years *vs.* ≥ 3 years) | **18** |

**Supplementary Table S1: The ICD codes of included ADs in the study**

| Diagnosis | ICD-10-CM Codes | ICD-11-CM Codes |
|---|---|---|
| Crohn's disease | K50 | DD70 |
| Ulcerative colitis | K51 | DD71 |
| Type 1 diabetes | E10 | 5A10 |
| Multiple sclerosis | G35 | 8A40 |
| Myasthenia gravis | G70.0 | 8C60 |
| Polymyalgia rheumatica | M35.3 | FA22 |
| Rheumatoid arthritis | M05 | FA20 |
| Juvenile idiopathic arthritis | M08 | FA24 |
| Ankylosing spondylitis | M45 | FA92.0 |
| Psoriatic arthritis | L40.53 | FA21 |
| Sjögren's syndrome | M35.0 | 4A43.2 |
| Systemic lupus erythematosus | M32 | 4A40.0 |
| Antiphospholipid syndrome | D68.61 | 4A45 |
| Systemic sclerosis | M34 | 4A42 |
| Polymyositis | M33.2 | 4A41.1 |
| Dermatomyositis | M33 | 4A41.0 |
| Antineutrophil cytoplasmic antibody-associated vasculitis | I77.82 | 4A44.A |
| Behcet's diseases | M35.2 | 4A62 |
| Takayasu's arteritis | M31.4 | 4A44.1 |

**Supplementary Table S2: Classification criteria of autoimmune diseases**

| Autoimmune disease | Classification criteria |
|---|---|
| **Behcet's disease** | **International Criteria for Behçet's Disease, 2014**[1]<br>• Two points for either oral ulcers, genital ulcers, or ocular lesions<br>• One point for cutaneous, neurological or vascular involvement, or a positive pathergy test<br>• Score of at least four points needed for Behçet's syndrome diagnosis |
| **Takayasu arteritis** | **2022 American college of rheumatology/EULAR classification criteria for Takayasu arteritis**[2]<br>Absolute requirements<br>Age $\leq$ 60 years at time of diagnosis<br>Evidence of vasculitis on imaging<br>Additional clinical criteria<br>Female sex +1<br>Angina or ischemic cardiac pain +2<br>Arm or leg claudication +2<br>Vascular bruit +2<br>Reduced pulse in upper extremity +2<br>Carotid artery abnormality +2<br>Systolic blood pressure difference in arms > 20 mmHg +1<br>Additional imaging criteria<br>Number of affected arterial territories (select one)<br>One arterial territory +1<br>Two arterial territories +2<br>Three or more arterial territories +3<br>Symmetric involvement of paired arteries +1<br>Abdominal aorta involvement with renal or mesenteric involvement +3<br>Absolute requirements must be satisfied. Sum the scores for 10 items, if present. A score of > 5 points is needed for the classification of Takayasu arteritis. |
| **Polymyalgia rheumatica** | **2012 ACR/EULAR classification criteria for PMR**[3]<br>PMR classification criteria scoring algorithm-required criteria: Age 50 years or older, bilateral shoulder aching and abnormal CRP and/or ESR* |

| Item | Points without US (0-6) | Points with US (0-8) |
|---|---|---|
| Morning stiffness duration > 45 min | 2 | 2 |
| Hip pain or limited range of motion | 1 | 1 |
| Absence of RF or ACPA | 2 | 2 |
| Absence of other joint involvement | 1 | 1 |
| At least one shoulder with subdeltoid bursitis and/or | Not applicable | 1 |

| | | |
|---|---|---|
| biceps tenosynovitis and/or glenohumeral synovitis (either posterior or axillary) and at least one hip with synovitis and/or trochanteric bursitis | | |
| Both shoulders with subdeltoid bursitis, biceps tenosynovitis or glenohumeral synovitis | Not applicable | 1 |

*A score of 4 or more is categorized as PMR in the algorithm without US and a score of 5 or more is categorized as PMR in the algorithm with US.

**Sjögren's syndrome**

**2016 ACR/EULAR classification criteria for primary Sjogren's syndrome**[4]

**Inclusion criteria**: these criteria are applicable to any patient with at least one symptom of ocular or oral dryness (defined as a positive response to at least one of the following questions: Have you had daily, persistent, troublesome dry eyes for more than 3 months? Do you have a recurrent sensation of sand or gravel in the eyes? Do you use tear substitutes more than 3 times a day? Have you had a daily feeling of dry mouth for more than 3 months? Do you frequently drink liquids to aid in swallowing dry food?); or suspicion of SS from ESSDAI questionnaire (at least one domain with positive item)

**Exclusion criteria**: Prior diagnosis of any of the following conditions would exclude diagnosis of SS and participation in SS studies or therapeutic trials because of overlapping clinical features or interference with criteria tests: history of head and neck radiation treatment; active hepatitis C infection (with positive PCR); acquired immunodeficiency syndrome; sarcoidosis; amyloidosis; graft versus host disease; IgG4-related disease.

The classification of SS applies to any individual who meets the inclusion criteria, does not have any condition listed as exclusion criteria, and who has a score ≥ 4 when summing the weights from the following items:

| Item | Score |
|---|---|
| Labial salivary gland with focal lymphocytic sialadenitis and focus score ≥ 1. | 3 |
| Anti-SSA (Ro) + | 3 |
| Ocular staining score ≥ 5 (or van Bijsterfeld score ≥ 4) on at least one eye | 1 |
| Schirmer ≤ 5 mm/5min on at least one eye | 1 |
| Unstimulated whole saliva flow rate ≤ 0.1 ml/min | 1 |

**Antiphospholipid syndrome**

**2023 ACR/EULAR Antiphospholipid Syndrome Classification Criteria**[5]

**Entry Criteria**[a]
At least one documented[b] clinical criterion listed below (domains 1-6)
*plus*
A positive antiphospholipid antibody (aPL) test
(a lupus anticoagulant test, or moderate-to-high titers of anticardiolipin or anti-$\beta_2$-glycoprotein-I antibodies [IgG or IgM])
within three years[b] of the clinical criterion

↓

**If absent, do not attempt to classify as APS - If present, apply additive criteria**

↓

**Additive clinical and laboratory criteria**[a]
Do not count a clinical criterion if there is an equally or more likely explanation than APS.
Within each domain, only count the highest weighted criterion towards the total score.

| Clinical domains and criteria | Weight | | Weight |
|---|---|---|---|
| **D1. Macrovascular (Venous Thromboembolism [VTE])** | | **D2. Macrovascular (Arterial Thrombosis [AT])** | |
| VTE with a high-risk VTE profile[c] | 1 | AT with a high-risk CVD profile[c] | 2 |
| VTE without a high-risk VTE profile[c] | 3 | AT without a high-risk CVD profile[c] | 4 |
| **D3. Microvascular** | | **D4. Obstetric** | |
| Suspected (one or more of the following) | 2 | ≥3 Consecutive pre-fetal (<10w) and/or early fetal (10w 0d -15w 6d) deaths | 1 |
| Livedo racemosa (exam) | | | |
| Livedoid vasculopathy lesions (exam) | | | |
| Acute/chronic aPL-nephropathy (exam or lab) | | Fetal death (16w 0d – 33w 6d) in the absence of pre-eclampsia (PEC) with severe features or placental insufficiency (PI) with severe features | 1 |
| Pulmonary hemorrhage (symptoms and imaging) | | | |
| Established (one of more of the following) | 5 | | |
| Livedoid vasculopathy (pathology[d]) | | | |
| Acute/chronic aPL-nephropathy (pathology[d]) | | PEC with severe features (<34w 0d) or PI with severe features (<34w 0d) with/without fetal death | 3 |
| Pulmonary hemorrhage (BAL or pathology[d]) | | | |
| Myocardial disease (imaging or pathology) | | PEC with severe features (<34w 0d) and PI with severe features (<34w 0d) with/without fetal death | 4 |
| Adrenal hemorrhage (imaging or pathology) | | | |
| **D5. Cardiac Valve** | | **D6. Hematology** | |
| Thickening | 2 | Thrombocytopenia (lowest 20-130x10$^9$/L) | 2 |
| Vegetation | 4 | | |
| **Laboratory (aPL) domains and criteria**[e] | **Weight** | | |
| **D7. aPL test by coagulation-based functional assay (lupus anticoagulant test [LAC])** | | **D8. aPL test by solid phase assay (anti-cardiolipin antibody [aCL] ELISA and/or anti-$\beta_2$-glycoprotein-I antibody [a$\beta_2$GPI] ELISA [persistent])** | |
| Positive LAC (single – one time) | 1 | Moderate or high positive (IgM) (aCL and/or a$\beta_2$GPI) | 1 |
| Positive LAC (persistent) | 5 | Moderate positive (IgG) (aCL and/or a$\beta_2$GPI) | 4 |
| | | High positive (IgG) (aCL or a$\beta_2$GPI) | 5 |
| | | High positive (IgG) (aCL and a$\beta_2$GPI) | 7 |

↓

**TOTAL SCORE**
Classify as Antiphospholipid Syndrome for research purposes if there are
at least 3 points from clinical domains AND at least 3 points from laboratory domains

**Rheumatoid arthritis**

**2010 ACR/EULAR rheumatoid arthritis classification criteria**[6]

Target population (who should be tested?): patients who

(1) have at least one joint with definite clinical synovitis (swelling)*

(2) with the synovitis not better explained by another disease†

Classification criteria for RA (score-based algorithm: add score of categories A–D a score of ≥ 6/10 is needed for classification of a patient as having definite RA)‡

A. Joint involvement§

1 large joint¶ 0

2−10 large joints 1

1−3 small joints (with or without involvement of large joints)** 2

4–10 small joints (with or without involvement of large joints) 3

> 10 joints (at least one small joint)[††] 5

B. Serology (at least 1 test result is needed for classification)[‡]

Negative RF and negative ACPA 0

Low-positive RF or low-positive ACPA 2

High-positive RF or high-positive ACPA 3

C. Acute-phase reactants (at least one test result is needed for classification)[§§]

Normal CRP and normal ESR 0 0

Abnormal CRP or normal ESR 1 1

D. Duration of symptoms[¶¶]

< 6 weeks 0

⩾ 6 weeks 1

*The criteria are aimed at classification of newly presenting patients. In addition, patients with erosive disease typical of RA with a history compatible with prior fulfilment of the 2010 criteria should be classified as having RA. Patients with long-standing disease, including those whose disease is inactive (with or without treatment) who, based on retrospectively available data, have previously fulfilled the 2010 criteria should be classified as having RA.

†Differential diagnoses differ in patients with different presentations, but may include conditions such as systemic lupus erythematosus, psoriatic arthritis and gout. If it is unclear about the relevant differential diagnoses to consider, an expert rheumatologist should be consulted.

‡Although patients with a score of less than 6/10 are not classifiable as having RA, their status can be reassessed and the criteria might be fulfilled cumulatively over time.

§Joint involvement refers to any swollen or tender joint on examination, which may be confirmed by imaging evidence of synovitis. Distal interphalangeal joints, first carpometacarpal joints and first metatarsophalangeal joints are excluded from assessment. Categories of joint distribution are classified according to the location and number of involved joints, with placement into the highest category possible based on the pattern of joint involvement.

¶ 'Large joints' refers to shoulders, elbows, hips, knees and ankles.

** 'Small joints' refers to the metacarpophalangeal joints, proximal interphalangeal joints, second to fifth metatarsophalangeal joints, thumb interphalangeal joints and wrists.

††In this category, at least one of the involved joints must be a small joint; the other joints can include any combination of large and additional small joints, as well as other joints not specifically listed elsewhere (*e.g.*, temporomandibular, acromioclavicular, sternoclavicular, *etc.*).

‡‡Negative refers to international unit (IU) values that are less than or equal to the upper limit of normal (ULN) for the laboratory and assay; low-positive refers to IU values that are higher than the ULN but three of less times the ULN for the laboratory and assay; high-positive refers to IU values that are more than three

| | |
|---|---|
| | times the ULN for the laboratory and assay. When rheumatoid factor (RF) information is only available as positive or negative, a positive result should be scored as low-positive for RF.<br>§§Normal/abnormal is determined by local laboratory standards.<br>¶¶Duration of symptoms refers to patient self-report of the duration of signs or symptoms of synovitis (*e.g.*, pain, swelling, tenderness) of joints that are clinically involved at the time of assessment, regardless of treatment status. |
| **Ankylosing spondylitis** | **Modified New York diagnostic criteria for ankylosing spondylitis[7]**<br>Clinical criteria<br>(1) Low back pain persisting for ≥ 3 months, reduced by exercise and not relieved by rest<br>(2) Limited motion in the lumbar spine in coronal and sagittal planes<br>(3) Limited chest expansion compared with normal values for age and sex<br>Radiologic criterion<br>Grade 3-4 unilateral or grade 2-4 bilateral sacroiliitis<br>Definite ankylosing spondylitis: The radiologic criterion and ≥ 1 of clinical criteria are fulfilled.<br>Probable ankylosing spondylitis: Three clinical criteria are fulfilled or the radiologic criterion alone is fulfilled. |
| **Systemic lupus erythematosus** | **2019 EULAR/ACR classification criteria for SLE[8]** |

**Entry criterion**

Antinuclear antibodies (ANA) at a titer of ≥1:80 on HEp-2 cells or an equivalent positive test (ever)

↓

If absent, do not classify as SLE

If present, apply additive criteria

↓

**Additive criteria**

**Do not count a criterion if there is a more likely explanation than SLE.**

**Occurrence of a criterion on at least one occasion is sufficient.**

**SLE classification requires at least one clinical criterion and ≥10 points.**

**Criteria need not occur simultaneously.**

**Within each domain, only the highest weighted criterion is counted toward the total score§.**

| Clinical domains and criteria | Weight | Immunology domains and criteria | Weight |
|---|---|---|---|
| ***Constitutional*** | | ***Antiphospholipid antibodies*** | |
| Fever | 2 | Anti-cardiolipin antibodies OR | |
| ***Hematologic*** | | Anti-β2GP1 antibodies OR | |
| Leukopenia | 3 | Lupus anticoagulant | 2 |
| Thrombocytopenia | 4 | ***Complement proteins*** | |
| Autoimmune hemolysis | 4 | Low C3 OR low C4 | 3 |
| ***Neuropsychiatric*** | | Low C3 AND low C4 | 4 |
| Delirium | 2 | ***SLE-specific antibodies*** | |
| Psychosis | 3 | Anti-dsDNA antibody* OR | |
| Seizure | 5 | Anti-Smith antibody | 6 |
| ***Mucocutaneous*** | | | |
| Non-scarring alopecia | 2 | | |
| Oral ulcers | 2 | | |
| Subacute cutaneous OR discoid lupus | 4 | | |
| Acute cutaneous lupus | 6 | | |
| ***Serosal*** | | | |
| Pleural or pericardial effusion | 5 | | |
| Acute pericarditis | 6 | | |
| ***Musculoskeletal*** | | | |
| Joint involvement | 6 | | |
| ***Renal*** | | | |
| Proteinuria >0.5g/24h | 4 | | |
| Renal biopsy Class II or V lupus nephritis | 8 | | |
| Renal biopsy Class III or IV lupus nephritis | 10 | | |

**Total score:**

↓

**Classify as Systemic Lupus Erythematosus with a score of 10 or more if entry criterion fulfilled.**

**Juvenile idiopathic arthritis**

**2019 PRINTO Classification Criteria for JIA**[9]

Fever of unknown origin (excluding infectious, neoplastic, autoimmune, or monogenic autoinflammatory diseases) that is documented to be daily (quotidian; fever that rises to ⩾ 39°C once a day and returns to ⩽ 37 °C between fever peaks) for at least 3 consecutive days and reoccurring over a duration of at least 2 weeks and accompanied by 2 major criteria OR 1 major criterion and 2 minor criteria.

Major criteria are (1) evanescent (nonfixed) erythematous rash; and (2) arthritis.

Minor criteria are (1) generalized lymph node enlargement and/or hepatomegaly

| | |
|---|---|
| | and/or splenomegaly; (2) serositis; (3) arthralgia lasting 2 weeks or longer (in the absence of arthritis); and (4) leukocytosis (≥ 15,000/$mm^3$) with neutrophilia. |

**Supplementary Table S3:** Characteristics of cGVHD in patients with pre-existing AD

| Characteristics | Patients with cGVHD (*n* = 29) |
|---|---|
| Time from HSCT to cGVHD (m) | 11 (5.5-7.5) |
| NIH severity (%) | |
| Mild | 22 (75.9%) |
| Moderate | 5 (17.2%) |
| Severe | 2 (6.9%) |
| Organ involvement | |
| Skin | 14 (48.3%) |
| Lung | 3 (10.3%) |
| Mouth | 4 (13.8%) |
| Liver | 0 |
| Eyes | 14 (48.3%) |
| Gastrointestinal | 1 (3.4%) |
| Joint/Fascia | 1 (3.4%) |
| Kidney | 1 (3.4%) |

Data are presented as *n* (%) or median (IQR). AD: autoimmune disease; HSCT: hematopoietic stem cell transplantation; GVHD: graft versus host disease; NIH: National Institutes of Health.

**Supplementary Table S4:** Univariable and multivariable analysis of prognostic factors for OS

| Variable | Univariable COX | | Multivariable COX | |
|---|---|---|---|---|
| | HR (95% CI) | *P* | HR (95% CI) | *P* |
| Age ≥ 60 years | 1.639 (0.583-4.610) | 0.349 | | |
| Female *vs.* male | 0.733 (0.397-1.352) | 0.320 | | |
| cGVHD | | | | |
| None | Reference | | | |
| Limited | 0.884 (0.387-2.022) | 0.771 | | |
| Extensive | 0.330 (0.105-1.041) | 0.058 | | |
| III-IV aGVHD | 3.573 (1.643-7.770) | 0.001 | 3.036 (1.383-6.663) | 0.006 |
| MRD *vs.* others | 0.409 (0.146-1.148) | 0.090 | | |
| CMV infection | 2.436 (1.193-4.973) | 0.014 | 2.189 (1.063-4.508) | 0.034 |
| EBV infection | 1.056 (0.376-2.965) | 0.917 | | |
| Bu/Cy+ATG *vs.* others | 1.596 (0.800-3.185) | 0.185 | | |
| ADs *vs.* controls | 1.116 (0.547-2.280) | 0.763 | | |

OS: overall survival; HR: hazard ratio; CI: confidence interval; cGVHD: chronic graft versus host disease; MRD: matched related donor; CMV: cytomegalovirus; EBV: Epstein-Barr virus.

**Supplementary Table S5:** Univariable and multivariable analysis of prognostic factors for DFS

| Variable | Univariable COX | | Multivariable COX | |
|---|---|---|---|---|
| | **HR (95% CI)** | ***P*** | **HR (95% CI)** | ***P*** |
| Age ≥ 60 years | 1.388 (0.497-3.875) | 0.531 | | |
| Female *vs.* male | 0.669 (0.377-1.185) | 0.168 | | |
| cGVHD | | | | |
| None | Reference | | | |
| Limited | 0.803 (0.368-1.754) | 0.582 | | |
| Extensive | 0.522 (0.201-1.353) | 0.181 | | |
| III-IV aGVHD | 3.575 (1.721-7.430) | 0.001 | 3.066 (1.462-6.430) | 0.003 |
| MRD *vs.* others | 0.448 (0.177-1.133) | 0.090 | 2.058 (1.059-4.002) | 0.033 |
| CMV infection | 2.291 (1.189-4.417) | 0.013 | | |
| EBV infection | 1.167 (0.461-2.951) | 0.745 | | |
| Bu/Cy+ATG *vs.* others | 1.721 (0.893-3.316) | 0.105 | | |
| ADs *vs.* controls | 1.207 (0.626-2.328) | 0.575 | | |

OS: overall survival; HR: hazard ratio; CI: confidence interval; cGVHD: chronic graft versus host disease; MRD: matched related donor; CMV: cytomegalovirus; EBV: Epstein-Barr virus.

**Supplementary Table S6:** Univariable and multivariable analysis of risk factors for NRM

| Variable | Univariable COX | | Multivariable COX | |
|---|---|---|---|---|
| | **HR (95% CI)** | ***P*** | **HR (95% CI)** | ***P*** |
| Age ≥ 60 years | 2.278 (0.793-6.548) | 0.126 | | |
| Female *vs.* male | 0.806 (0.393-1.651) | 0.555 | | |
| cGVHD | | | | |
| None | Reference | | | |
| Limited | 0.855 (0.320-2.284) | 0.755 | | |
| Extensive | 0.460 (0.133-1.589) | 0.219 | | |
| III-IV aGVHD | 3.663 (1.491-9.001) | 0.005 | 3.131 (1.262-7.764) | 0.014 |
| MRD *vs.* others | 0.426 (0.129-1.404) | 0.161 | 2.310 (0.982-5.436) | 0.055 |
| CMV infection | 2.559 (1.097-5.968) | 0.030 | | |
| EBV infection | 1.047 (0.317-3.451) | 0.940 | | |
| Bu/Cy+ATG *vs.* others | 1.379 (0.632-3.011) | 0.420 | | |
| ADs *vs.* controls | 1.435 (0.656-3.136) | 0.365 | | |

OS: overall survival; HR: hazard ratio; CI: confidence interval; cGVHD: chronic graft versus host disease; MRD: matched related donor; CMV: cytomegalovirus; EBV: Epstein-Barr virus.

**Supplementary Table S7:** Univariable and multivariable analysis of risk factors for CIR

| Variable | Univariable COX | | Multivariable COX | |
|---|---|---|---|---|
| | **HR (95% CI)** | ***P*** | **HR (95% CI)** | ***P*** |

| | | | | |
|---|---|---|---|---|
| Age ≥ 60 years | 0.045 (0-212.595) | 0.472 | | |
| Female *vs.* male | 0.485 (0.185-1.275) | 0.142 | | |
| cGVHD | | | | |
| None | Reference | | | |
| Limited | 0.737 (0.203-2.679) | 0.643 | | |
| Extensive | 0.622 (0.139-2.782) | 0.535 | | |
| III-IV aGVHD | 3.588 (1.023-12.579) | 0.046 | 3.588 (1.023-12.579) | 0.046 |
| MRD *vs.* others | 0.496 (0.113-2.170) | 0.352 | | |
| CMV infection | 1.888 (0.665-5.363) | 0.233 | | |
| EBV infection | 1.328 (0.303-5.817) | 0.706 | | |
| Bu/Cy+ATG *vs.* others | 2.822 (0.811-9.822) | 0.103 | | |
| ADs *vs.* controls | 0.762 (0.219-2.659) | 0.670 | | |

OS: overall survival; HR: hazard ratio; CI: confidence interval; cGVHD: chronic graft versus host disease; MRD: matched related donor; CMV: cytomegalovirus; EBV: Epstein-Barr virus.

**Supplementary Table S8:** Individual patient characteristics and outcome

| No. | AD type | AD duration (y) | HM | Sex | Age | Pre-transplant AD activity | Pre-transplant AD medication | Remission status | Time to CR (m) | CR duration (m) | AD-related interventions post-transplant |
|---|---|---|---|---|---|---|---|---|---|---|---|
| 1 | BD | 1 | MDS | M | 27 | Active disease | Prednisone, Mesalamine, Thalidomide | | | | |
| 2 | BD | 14 | MDS | M | 54 | Controlled on medication | Methylprednisolone, Colchicine | PR | | | Immunosuppression |
| 3 | BD | 5 | MDS | M | 54 | Controlled on medication | Prednisone, Cyclosporine | | | | |
| 4 | BD | 23 | MDS | F | 42 | Controlled on medication | Thalidomide | CR | 6 | 24 | Corticosteroids + Immunosuppression |
| 5 | BD | 3 | MDS | F | 25 | Controlled on medication | Thalidomide | CR | 6 | 10 | |
| 6 | BD | 2 | NHL | M | 42 | Controlled on medication | Thalidomide | CR | 6 | 3 | |
| 7 | BD | 6 | AML | F | 39 | Active disease | | CR | 6 | 5 | |
| 8 | Arteritis | 9 | AML | F | 52 | Controlled on medication | Prednisone, Immunosuppression | CR | 6 | 46 | |
| 9 | SS+RA | 10 | AML | F | 41 | Active disease | | NR | | | Biologics + DMARDS |
| 10 | SS | 6 | MDS | F | 47 | Controlled on medication | Prednisone, Immunosuppression | CR | 6 | 59 | |
| 11 | SS | 1 | MDS | F | 52 | Controlled on | Prednisone, | CR | 6 | 22 | |

| | | | | | | | | | | | |
|---|---|---|---|---|---|---|---|---|---|---|---|
| | | | | | | medication | Hydroxychloroquine, Total glucosides of paeonia | | | | |
| 12 | BD | 2 | MDS | F | 42 | Active disease | | CR | 3 | 0 | |
| 13 | RA | 4 | AML | F | 30 | Controlled on medication | Prednisone, MTX, Hydroxychloroquine, Leflunomide | | | | |
| 14 | RA | 10 | ALL | F | 47 | Controlled on medication | NSAIDs | | | | |
| 15 | RA | 13 | AML | F | 47 | Active disease | Hydroxychloroquine, Leflunomide, Etanercept | PR | | | DMARDS |
| 16 | RA | 6 | AML | F | 57 | Controlled on medication | Methylprednisolone, Hydroxychloroquine, Sulfasalazine | CR | 7 | 8 | |
| 17 | AS | 30 | AML | M | 47 | Controlled on medication | NSAIDs | CR | 6 | 0 | |
| 18 | AS | 30 | B-ALL | M | 50 | Active disease | Sulfasalazine | CR | 8 | 136 | |
| 19 | AS | 2 | B-ALL | M | 21 | Active disease | NSAIDs | NR | | | Biologics + DMARDS |
| 20 | AS | 4 | AML | M | 24 | Controlled on medication | Thalidomide | CR | 6 | 102 | |
| 21 | AS | 1 | B-ALL | F | 23 | Controlled on medication | NSAIDs | | | | |
| 22 | AS | 5 | B-ALL | M | 24 | Controlled on | NSAIDs | | | | |

| | | | | | | | | | | | |
|---|---|---|---|---|---|---|---|---|---|---|---|
| | | | | | | medication | | | | | |
| 23 | AS | 1 | B-ALL | M | 28 | Active disease | | PR | | | DMARDS |
| 24 | AS | 4 | AML | M | 16 | Active disease | NSAIDs, Sulfasalazine, Etanercept | CR | 8 | 40 | |
| 25 | AS | 5 | NHL | F | 37 | Controlled on medication | NSAIDs | CR | 9 | 28 | |
| 26 | AS | 20 | CMML | M | 61 | Controlled on medication | Sulfasalazine | NR | | | DMARDS |
| 27 | AS | 8 | AML | M | 53 | Active disease | NSAIDs | NR | | | DMARDS |
| 28 | AS | 7 | B-ALL | M | 25 | Controlled on medication | NSAIDs | CR | 9 | 38 | |
| 29 | AS | 5 | MDS | M | 64 | Controlled on medication | NSAIDs | | | | |
| 30 | AS | 9 | AML | F | 29 | Controlled on medication | Etanercept, Thalidomide, Prednisone | CR | 9 | 52 | |
| 31 | SLE | 2 | MDS | M | 53 | Controlled on medication | Prednisone | CR | 6 | 138 | |
| 32 | SLE | 1 | B-ALL | F | 19 | Controlled on medication | Prednisone | CR | 6 | 23 | |
| 33 | SLE | 9 | AML | F | 44 | Controlled on medication | Prednisone, danazol, Cyclosporine, Hydroxychloroquine | CR | 6 | 18 | |
| 34 | SLE | 7 | AML | F | 36 | Controlled on | Prednisone, CTX, | CR | 6 | 5 | |

| | | | | | | | | | | | |
|---|---|---|---|---|---|---|---|---|---|---|---|
| | | | | | | medication | Hydroxychloroquine | | | | |
| 35 | SLE+ APS | 1 | CMML | F | 46 | Active disease | Prednisone, Hydroxychloroquine | | | | |
| 36 | PMR | 1 | ALL | F | 45 | Controlled on medication | Prednisone | CR | 8 | 169 | |
| 37 | PMR | 1 | AML | F | 57 | Controlled on medication | Prednisone | | | | |
| 38 | RA | 5 | B-ALL | F | 54 | Controlled on medication | MTX, Leflunomide, Total glucosides of paeonia | CR | 6 | 3 | |
| 39 | JIA | 1 | B-ALL | F | 16 | Active disease | Prednisone, immunosuppression | | | | |
| 40 | APS | 3 | B-ALL | F | 26 | Controlled on medication | Prednisone, Hydroxychloroquine | CR | 7 | 145 | |
| 41 | RA | 1 | AML | M | 63 | Active disease | | NR | | | DMARDS |

AD: autoimmune disease; HM: Hematological malignancies; AML: acute myeloid leukemia; ALL: acute lymphoblastic leukemia; MDS: myelodysplastic syndrome; CMML: chronic myelomonocytic leukemia; NHL: non-Hodgkin lymphoma; M: male; F: female; BD: Behcet's diseases; PMR: polymyalgia rheumatica; SS: Sjogren's syndrome; APS: antiphospholipid syndrome; RA: rheumatoid arthritis; AS: ankylosing spondylitis; SLE: systemic lupus erythematosus; JIA: juvenile idiopathic arthritis; CR: complete remission; PR: partial remission; NR: no remission; MTX: Methotrexate; CTX: Cyclophosphamide; NSAIDs: Non-steroidal anti-inflammatory drugs; DMARDS: disease-modifying antirheumatic drugs.

**Supplementary Table S9:** Time to complete remission across different AD types

| ADs type | Time from allo-HSCT to CR (m) |
|---|---|
| AS (*n* = 7) | 8 (6-9) |
| BD (*n* = 5) | 6 (4.5-6) |
| SLE (*n* = 4) | 6 (6-6) |
| Others (*n* = 7) | 6 (6-7) |

Data are presented as median (IQR). AD: autoimmune disease; BD: Behcet's diseases; AS: ankylosing spondylitis; SLE: systemic lupus erythematosus; CR: complete remission.

**Supplementary Table S10:** Association between the development of cGVHD and the achievement of complete remission of the pre-existing AD

| Item | AD not in CR | AD in CR | *P* value |
|---|---|---|---|
| No cGVHD | 2 (25.0%) | 9 (39.1%) | 0.676 |
| cGVHD | 6 (75.0%) | 14 (60.9%) | |

Data are presented as *n* (%). AD: autoimmune disease; CR: complete remission; cGVHD: chronic graft versus host disease.

**Supplementary Table S11:** Transplant outcomes across major autoimmune disease subtypes

| Characteristics | AS (*n* = 14) | BD (*n* = 8) | RA (*n* = 6) | Others (*n* = 13) |
|---|---|---|---|---|
| WBC engraftment (days) | 13.5 (11.8-15.3) | 15 (12.3-17) | 13.5 (12-15.3) | 14 (11-15) |
| PLT engraftment (days) | 18 (13.8-24.5) | 18 (16-19.5) | 17.5 (10.5-22) | 14.5 (12.3-19) |
| III-IV aGVHD | 1 (7.1%) | 2 (25%) | 0 | 1 (7.7%) |
| cGVHD | 12 (85.7%) | 6 (75%) | 1 (16.7%) | 10 (76.9%) |
| CMV viremia | 3 (21.4%) | 5 (62.5%) | 2 (33.3%) | 5 (38.5%) |
| EBV viremia | 1 (7.1%) | 2 (25%) | 1 (16.7%) | 2 (15.4%) |
| 3-year DFS (%) | 71.4 (95% CI 51.3-99.5) | 72.9 (95% CI 46.8-100) | 60.0 (95% CI 29.3-100) | 69.2 (95% CI 48.2-99.5) |
| 3-year OS (%) | 77.9 (95% CI 58.7-100) | 72.9 (95% CI 46.8-100) | 60.0 (95% CI 29.3-100) | 75.0 (95% CI 54.1-100) |
| 3-year NRM (%) | 14.3 (95% CI 2.08-37.6) | 27.1 (95% CI 2.94-61.6) | 40.0 (95% CI 3.05-78.6) | 23.1 (95% CI 5.08-48.6) |
| 3-year CIR (%) | 14.3 (95% CI 2.09-37.6) | 0 | 0 | 7.69 (95% CI 0.41-30.3) |
| Complete remission of AD | 63.6% (7/11) | 83.3% (5/6) | 50% (2/4) | 90% (9/10) |

Data are presented as *n* (%), median (IQR), or median (95% CI). AS: ankylosing spondylitis; BD: Behcet's diseases; RA: rheumatoid arthritis; WBC: white blood cell; PLT: platelet; aGVHD: acute graft versus host disease; cGVHD: chronic graft versus host disease; CMV: cytomegalovirus; EBV: Epstein-Barr virus; OS: overall survival; DFS: disease free survival; NRM: nonrelapse mortality; CIR: cumulative incidence of relapse; ADs: autoimmune diseases.

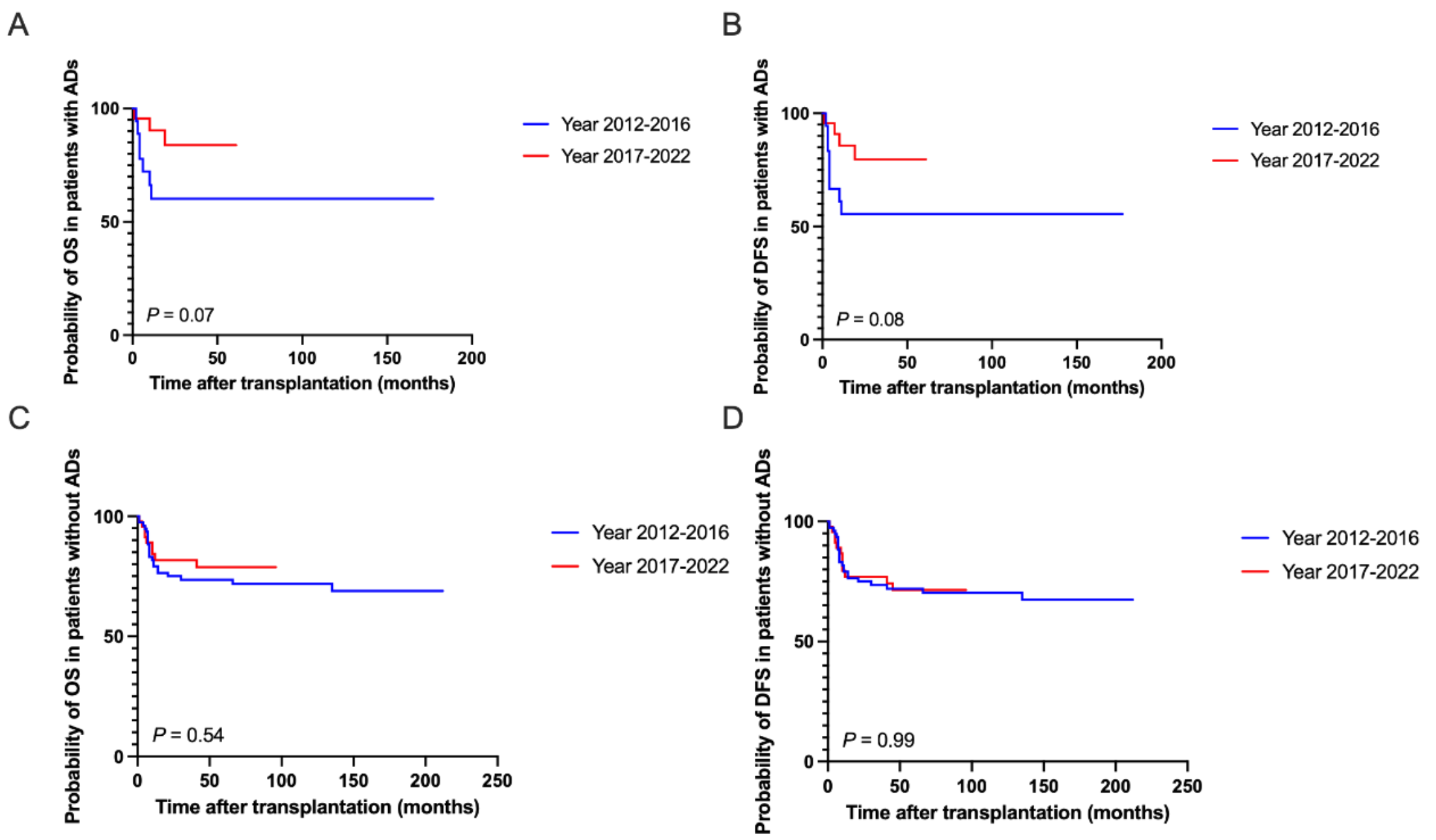


**Supplementary Figure S1: Comparison of survival probabilities stratified by transplant year period (2012-2016 *vs.* 2017-2022).** The rate of (A) OS and (B) DFS in patients with ADs. The rate of (C) OS and (D) DFS in patients without ADs. AD: autoimmune disease; OS: overall survival; DFS: disease free survival.

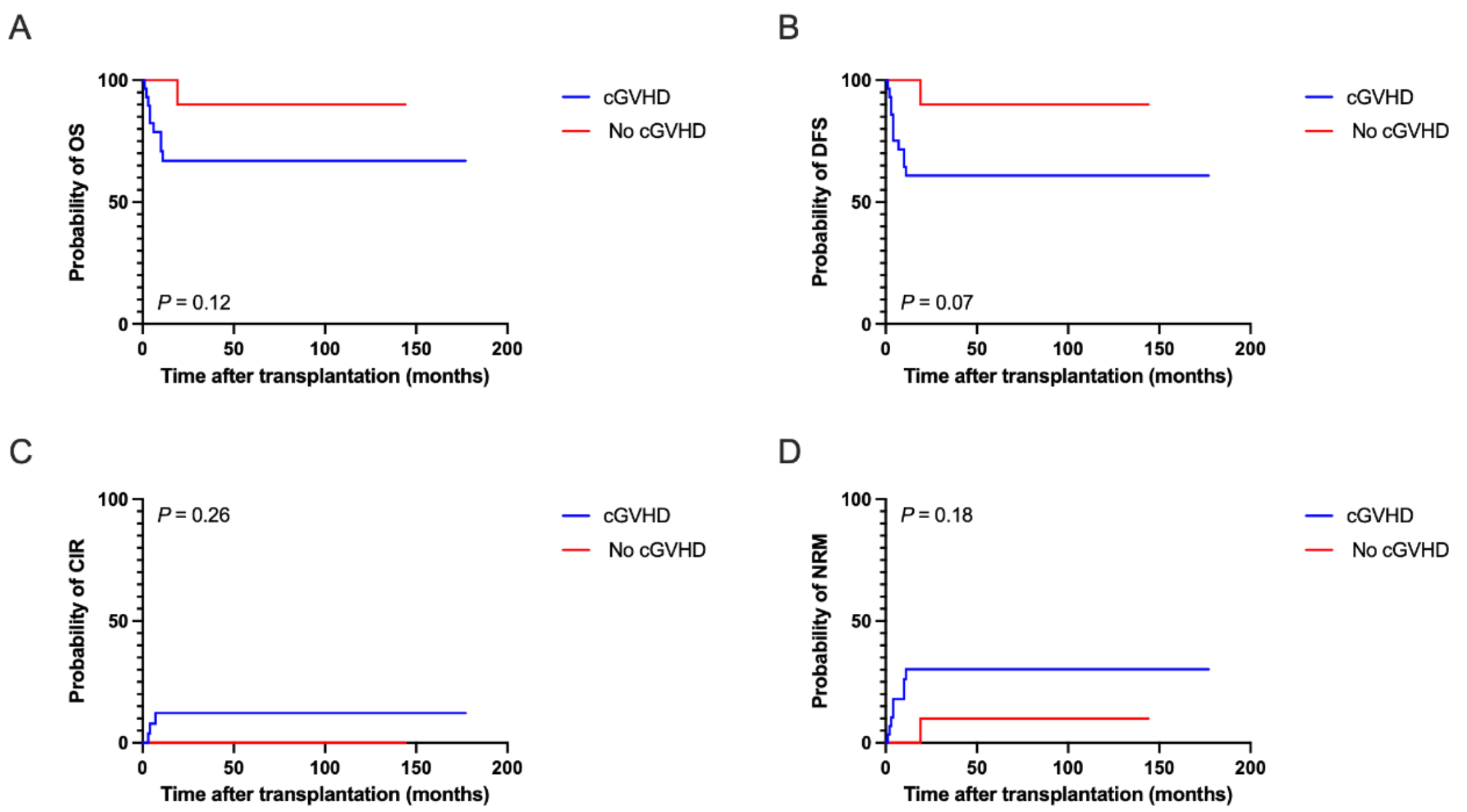


**Supplementary Figure S2: Association between chronic GVHD and key transplant outcomes in patients with hematological malignancies with pre-existing AD.** The rate of (A) OS and (B) DFS. (C) The incidence of relapse. (D) The incidence of NRM. AD: autoimmune disease; OS: overall survival; DFS: disease free survival; NRM: nonrelapse mortality.

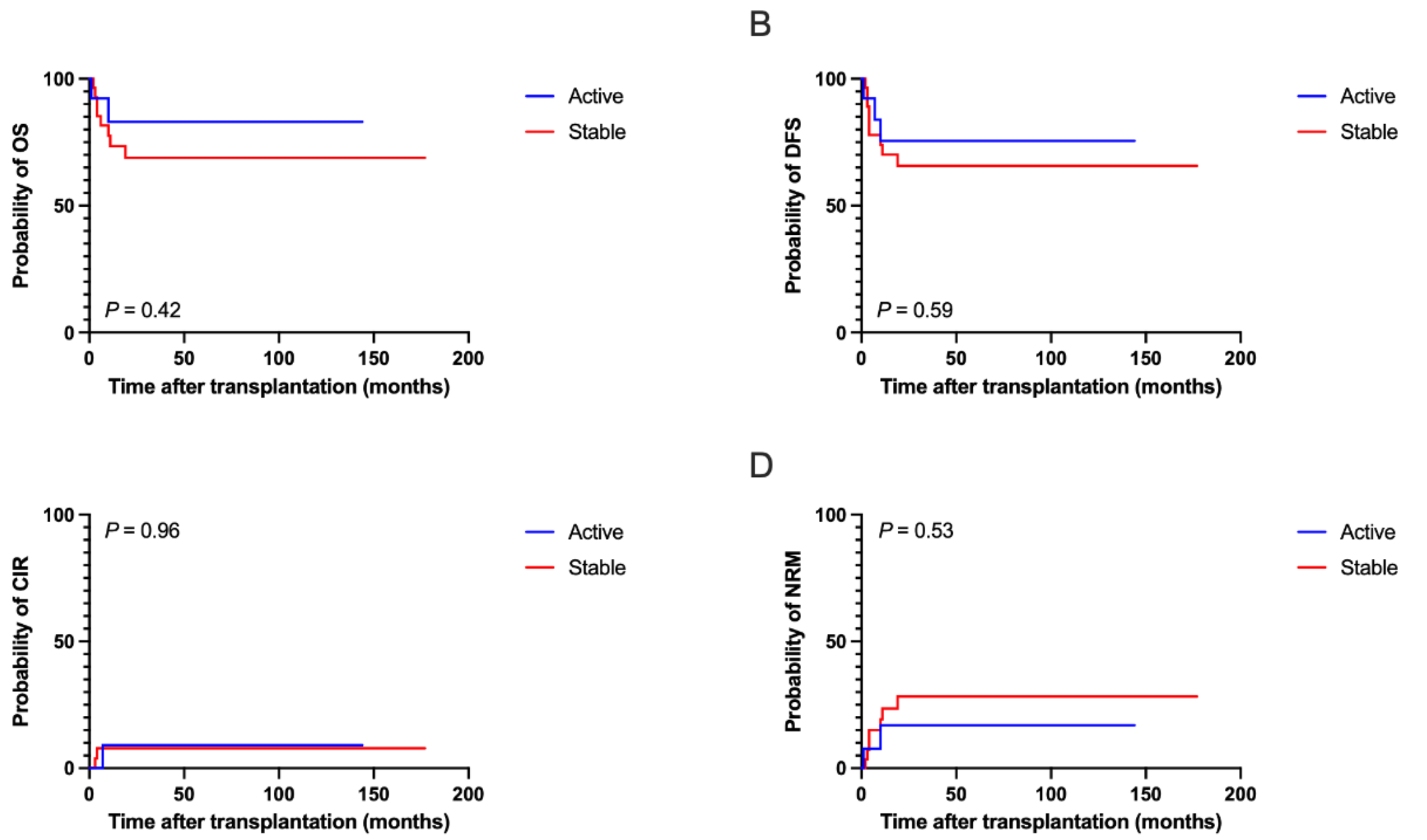


**Supplementary Figure S3: Association between ADs severity and key transplant outcomes in patients with hematological malignancies with pre-existing AD (active *vs.* stable).** The rate of (A) OS and (B) DFS. (C) The incidence of relapse. (D) The incidence of NRM. AD: autoimmune disease; OS: overall survival; DFS: disease free survival; NRM: nonrelapse mortality.

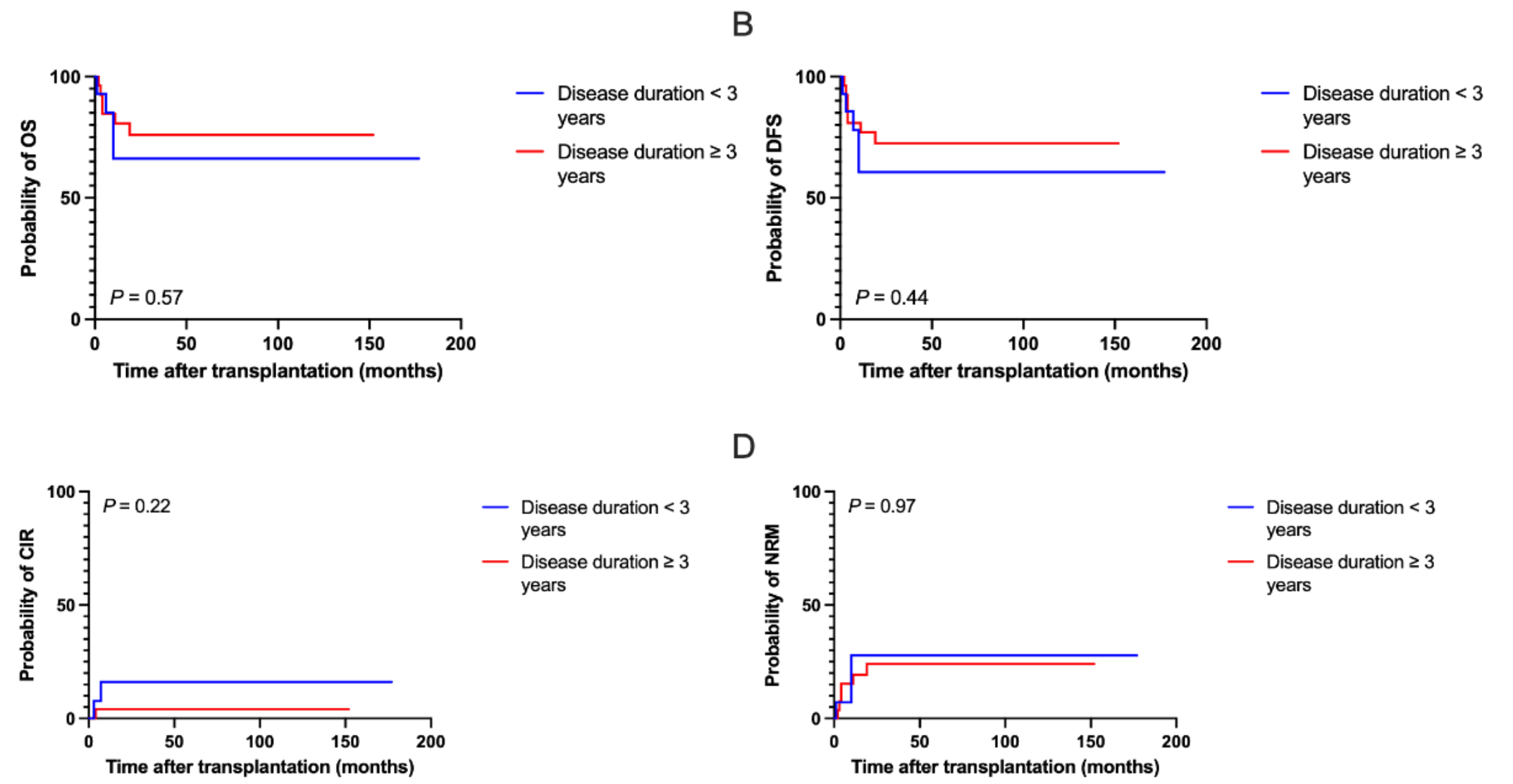


**Supplementary Figure S4: Association between ADs duration and key transplant outcomes in patients with hematological malignancies with pre-existing AD (disease duration < 3 years *vs.* ≥ 3 years).** The rate of (A) OS and (B) DFS. (C) The incidence of relapse. (D) The incidence of NRM. AD: autoimmune disease; OS: overall survival; DFS: disease free survival; NRM: nonrelapse mortality.

## References

1. International Team for the Revision of the International Criteria for Behçet's Disease (ITR-ICBD). The International Criteria for Behçet's Disease (ICBD): a collaborative study of 27 countries on the sensitivity and specificity of the new criteria. *J Eur Acad Dermatol Venereol* 2014;28(3):338–347.

2. Grayson PC, Ponte C, Suppiah R, Robson JC, Gribbons KB, Judge A, *et al*. 2022 American College of Rheumatology/EULAR classification criteria for Takayasu arteritis. *Ann Rheum Dis* 2022;81(12):1654–1660.

3. Dasgupta B, Cimmino MA, Maradit-Kremers H, Schmidt WA, Schirmer M, Salvarani C, *et al*. 2012 provisional classification criteria for polymyalgia rheumatica: a European League Against Rheumatism/American College of Rheumatology collaborative initiative. *Ann Rheum Dis* 2012;71(4):484–492.

4. Shiboski CH, Shiboski SC, Seror R, Criswell LA, Labetoulle M, Lietman TM, *et al*. 2016 American college of rheumatology/European league against rheumatism classification criteria for primary sjögren's syndrome: a consensus and data-driven methodology involving three international patient cohorts. *Arthritis Rheumatol* 2017;69(1):35–45.

5. Barbhaiya M, Zuily S, Naden R, Hendry A, Manneville F, Amigo M-C, *et al*. The 2023 ACR/EULAR antiphospholipid syndrome classification criteria. *Arthritis Rheumatol* 2023;75(10):1687–1702.

6. Aletaha D, Neogi T, Silman AJ, Funovits J, Felson DT, Bingham III CO, *et al*. 2010 Rheumatoid arthritis classification criteria: an American College of Rheumatology/European League Against Rheumatism collaborative initiative. *Arthritis Rheum* 2010;62(9):2569–2581.

7. van der Linden S, Valkenburg HA, Cats A. Evaluation of diagnostic criteria for ankylosing spondylitis. A proposal for modification of the New York criteria. *Arthritis Rheum* 1984;27(4):361–368.

8. Aringer M, Costenbader K, Daikh D, Brinks R, Mosca M, Ramsey-Goldman R, *et al*. 2019 European League Against Rheumatism/American College of Rheumatology classification criteria for systemic lupus erythematosus. *Arthritis Rheumatol* 2019;71(9):1400-1412.

9. Martini A, Ravelli A, Avcin T, Beresford MW, Burgos-Vargas R, Cuttica R, *et al*. Toward new classification criteria for juvenile idiopathic arthritis: first steps, pediatric rheumatology international trials organization international consensus. *J Rheumatol* 2019;46(2):190–197.
